# Analysis of publications on HPV genotype co-infection: a bibliometric study on existing research

**DOI:** 10.3389/fonc.2023.1218744

**Published:** 2023-07-24

**Authors:** Tianyi Bi, Yingxin Gong, Jiayin Mo, Yan Wang, Wenjie Qu, Yaping Wang, Wenqian Shi, Feifei Zhang, Long Sui, Yanyun Li

**Affiliations:** ^1^ Department of Gynecology and Obstetrics, Obstetrics and Gynecology Hospital of Fudan University, Shanghai, China; ^2^ Shanghai Key Laboratory of Female Reproductive Endocrine Related Diseases, Shanghai, China

**Keywords:** bibliometric analysis, human papillomavirus, co-infection, web of science, VOSviewer

## Abstract

**Purpose:**

To identify the bibliometric information of Human papillomavirus (HPV) genotype co-infection in certain literature database over the past two decades.

**Methods:**

Web of Science was used as the main database to identify all eligible articles focusing on HPV genotype co-infection at the date of October 16, 2022. From this journal database, we identified 463 articles on HPV genotype co-infection, conducted statistical analysis according to the author, journal, publication year and month, country or region, keyword and impact factor.

**Results:**

The articles included in our analysis were published between 1994 and 2022. The index of citations per year ranged from 170.4 to 13.1. These articles were from 78 countries or regions, with most publications from the United States (n = 73), followed by China (n = 65) and Italy (n = 50). The journal that contributed the most publications on HPV heterotypic gene co-infection was PLOS ONE with a total of 29 articles, followed by JOURNAL OF MEDICAL VIROLOGY (n = 28), INFECTIOUS AGENTS AND CANCER (n = 14) and JOURNAL OF CLINICAL VIROLOGY (n = 12). Among existing research in the field of HPV co-infection, we found that epidemiological distribution and infection mechanism has been the two major topics for scholars, and studies on detection methods for HPV multiple genotypes were also included.

**Conclusion:**

Over decades, epidemiological studies and mechanism investigationhas been the central topics when it comes to HPV genotypes co-infection. Studies on HPV co-infection remained relatively insufficient, mainly stays in qualitative level while detailed infection data and high quality literature publications were still lack of valuable discussion.

## Introduction

Human papillomavirus (HPV) is a common and highly invasive virus that causes diseases of the female population on the lower reproductive tract. *It is noteworthy that HPV can cause severe cervical cancer, and the World Health Organization (WHO) estimated a great prevalence of 604,000 new cases along with 342,000 deaths worldwide in 2020 (* 1). As a global public health concern, HPV has attracted extensive attention from researchers all over the world over decades. As a result, increasingly high-quality studies on HPV infection rate, pathogenesis and preventive vaccine has been popping up in recent years.

*As is known, HPV was a large family with more than 130 variants detected. In addition to single genotype infection, there are also a large number of patients showed more than one genotype joint infection (* 2)*. Such multiple genotype HPV infections are common in clinical practice, and their virulence has been proved to be relatively strong, which has a negative impact on the prognosis of HPV-associated diseases (* 3). However, the permutations of different genotypes showed more possibilities and hypothesis, studies on HPV co-infection with multiple genotypes are destined to be more complex than single genotype infection, and valueble research in this direction are still relatively insufficient.

At present, studies on HPV multiple infection mainly focused on epidemiological perspective. According to a study covered 137,943 gynecological outpatients by Guangdong Liao et al in 2020 reported that HPV 16/18 were likely to co-infected with HPV 31 but unlikely with HPV 52/58, and the co-infection of HPV 16 with HPV 31 also has a degree of presentation (4). In other parts of the world, HPV coinfection patterns can be different, with some cohort studies from Europe reporting higher levels of HPV16 and 31 co-infection (4–7). Similar studies are also underway in developing countries, for instance, a study on Tunisian crowd (8) and another on Zimbabwean crowd (9, 10) reported high co-infection prevalence of HPV16 and HPV35. There is no conclusive evidence as to how susceptible virus types differ between ethnic groups. Moreover, very few studies have focused on the mechanisms of HPV co-infection, and there is no consensus on whether there is a synergistic relationship between different HPV types.

Based on the research status mentioned above, it is surely necessary to conduct bibliometric studies on HPV co-infection. By statistical analysis of the similarities and differences among the existing publications, understanding the authors, sources, districts and research objectives of these literatures will help us to have a comprehensive and systematic understanding of this unfashionable subject on HPV infection. In this article, we conducted a bibliometric analysis of the literature related to HPV polygenotype infection over the past 20 years, explored the research trends and common patterns, tracked the research domain network, and predict the future direction on both theoretical and clinical research.

## Method

### Database and search strategy

The literature search was conducted on October 16, 2022 using the Web of Science core collecction as database on October 16, 2022, with the following search formula: TS=("human papillomavirus" OR HPV) AND TS=(co-infection OR coinfection OR "multiple infection" OR "mixed infections" OR “Polymicrobial Infection”) AND TS=(genotype OR genotypes OR genogroup). Inclusion criteria were as follows: (I) studies with HPV as the main topic; (II) English or Chinese written articles. Exclusion criteria were: (I) HPV was not the core of the study. (II)The study is about HPV co-infection with other viruses. In this article, a total of 463 publications from the web of science core collection were selected for bibliometric statistical analysis, published between 1994 and 2022. The author, journal, year and month of publication, country or region, and five-year infection factors were recorded. Article types were categorized into articles, reviews, abstract, meeting, clinical trial, case report.

### Data analysis

All search results and data critical to our analysis including citations and metrics were extracted from Web of Science on October 16, 2022. The complete document list was exported in the form of TXT file and imported into VosViewer for further analysis of co-occurrence atlas. WPS Excel 2022 was used to construct the tables and Originlab was used for bar charts and line charts visualization.

## Results

### Analysis of the number of publications and citations

The 463 articles on HPV genotype co-infection from WOS core collection included in our study were published from 1994 to 2022. An average of 32 references were cited by each articles, and the number of references cited ranging from 9 to 104 per paper, with a total of 14993 references. To explore which articles are more frequently cited with higher citation value, we ranked the publications with different citation frequencies. **Table 1** (2, 11–29) listed the top 20 most cited articles.

**Table 1:** The top 20 most cited articles on HPV co-infection (2, 11–29).

Number	Authors	Article Title	Source Title	Cited Reference	Times Cited by Search Date	Times Cited by Submit Date
1	Cuschieri KS, et al.	Multiple high risk HPV infections are common in cervical neoplasia and young women in a cervical screening population	JOURNAL OF CLINICAL PATHOLOGY	25	225	230
2	Chaturvedi AK, et al.	Human Papillomavirus Infection with Multiple Types: Pattern of Coinfection and Risk of Cervical Disease	JOURNAL OF INFECTIOUS DISEASES	29	196	203
3	Koskinen WJ, et al.	Prevalence and physical status of human papillomavirus in squamous cell carcinomas of the head and neck	INTERNATIONAL JOURNAL OF CANCER	29	164	164
4	Schmitt, M,et al.	Homogeneous amplification of genital human alpha papillomaviruses by PCR using novel broad-spectrum GP5+ and GP6+ primers	JOURNAL OF CLINICAL MICROBIOLOGY	19	148	154
5	Schmitt, M,et al.	Abundance of Multiple High-Risk Human Papillomavirus (HPV) Infections Found in Cervical Cells Analyzed by Use of an Ultrasensitive HPV Genotyping Assay	JOURNAL OF CLINICAL MICROBIOLOGY	26	141	145
6	van Doorn, LJ, et al.	Highly effective detection of human papillomavirus 16 and 18 DNA by a testing algorithm combining broad-spectrum and type-specific PCR	JOURNAL OF CLINICAL MICROBIOLOGY	37	138	139
7	Trottier, H,et al.	Type-specific duration of human papillomavirus infection: Implications for human papillomavirus screening and vaccination	JOURNAL OF INFECTIOUS DISEASES	44	131	133
8	Chan, PKS, et al.	High prevalence of human papillomavirus type 58 in Chinese women with cervical cancer and precancerous lesions	JOURNAL OF MEDICAL VIROLOGY	24	104	104
9	Nagpal, JK, et al.	Prevalence of high-risk human papilloma virus types and its association with p53 codon 72 polymorphism in tobacco addicted oral squamous cell carcinoma (OSCC) patients of eastern India	INTERNATIONAL JOURNAL OF CANCER	29	104	108
10	Vaccarella, S, et al.	Concurrent Infection with Multiple Human Papillomavirus Types: Pooled Analysis of the IARC HPV Prevalence Surveys	CANCER EPIDEMIOLOGY BIOMARKERS & PREVENTION	45	84	88
11	Chaturvedi, AK, et al.	Prevalence and clustering patterns of human papillomavirus genotypes in multiple infections	CANCER EPIDEMIOLOGY BIOMARKERS & PREVENTION	23	83	84
12	Cho, NH, et al.	Genotyping of 22 human papillomavirus types by DNA chip in Korean women: Comparison with cytologic diagnosis	AMERICAN JOURNAL OF OBSTETRICS AND GYNECOLOGY	25	79	79
13	Schmitt, M,et al.	Multiple Human Papillomavirus Infections with High Viral Loads Are Associated with Cervical Lesions but Do Not Differentiate Grades of Cervical Abnormalities	JOURNAL OF CLINICAL MICROBIOLOGY	34	76	81
14	Vajdic, CM, et al.	Anal human papillomavirus genotype diversity and co-infection in a community-based sample of homosexual men	SEXUALLY TRANSMITTED INFECTIONS	30	74	74
15	Tota, JE, et al.	Epidemiologic Approaches to Evaluating the Potential for Human Papillomavirus Type Replacement Postvaccination	AMERICAN JOURNAL OF EPIDEMIOLOGY	70	74	77
16	Rousseau, MC, et al.	Predictors of cervical coinfection with multiple human papillomavirus types	CANCER EPIDEMIOLOGY BIOMARKERS & PREVENTION	40	73	73
17	Baay, MFD, et al.	Human papillomavirus in a rural community in Zimbabwe: The impact of HIV co-infection on HPV genotype distribution	JOURNAL OF MEDICAL VIROLOGY	17	64	67
18	Dal Bello, B, et al.	Cervical Infections by Multiple Human Papillomavirus (HPV) Genotypes: Prevalence and Impact on the Risk of Precancerous Epithelial Lesions	JOURNAL OF MEDICAL VIROLOGY	36	62	66
19	Spinillo, A, et al.	Multiple human papillomavirus infection and high grade cervical intraepithelial neoplasia among women with cytological diagnosis of atypical squamous cells of undetermined significance or low grade squamous intraepithelial lesions	GYNECOLOGIC ONCOLOGY	23	61	65
20	Omland, T, et al.	Recurrent Respiratory Papillomatosis: HPV Genotypes and Risk of High-Grade Laryngeal Neoplasia	PLOS ONE	59	59	62

There were ten articles that had over 100 citations at the time of our search. The most cited article with a citation number of 225 was carried out by Cuschieri et al, published in 2004 on JOURNAL OF CLINICAL PATHOLOGY (2). This report investigated the diversity of multiple HPV types in a routine cervical screening population, and assessed its associations with cervical neoplasia. The main research topics among the top 20 cited publications of HPV co-infection can be summarized into the following four research topics: HPV co-infection and clinical pathogenicity (n=6), epidemiological study of HPV co-infection (n=5), infection mechanism of HPV co-infection (n=5), and laboratory detection method of HPV co-infection (n=3).

### Analysis of time distribution

**Figure 1:**
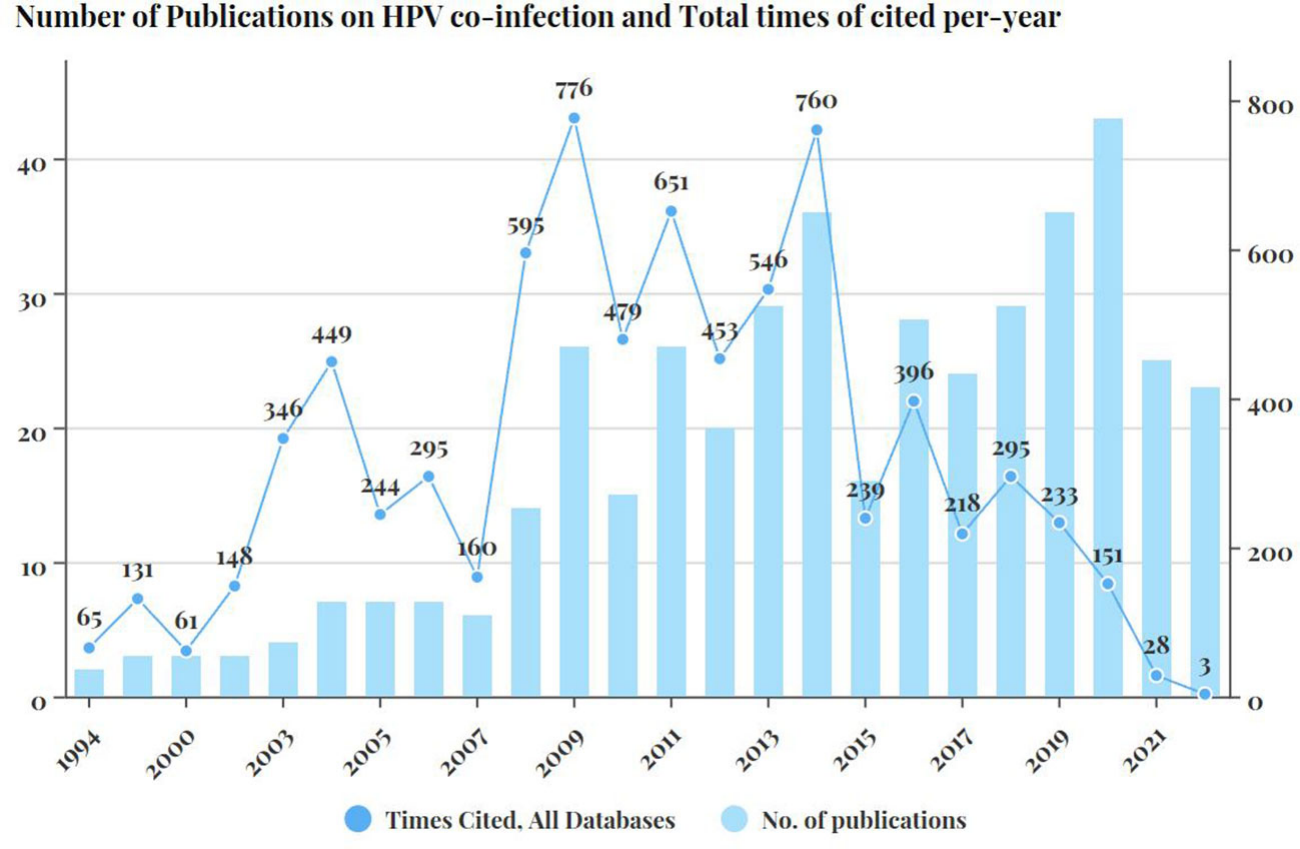
Number of publications on HPV co-infection and Total times of cited per-year.

The number of published articles also varied significantly according to publication years. **Figure 1** showed the total number of citations each year. The number of publications related to multiple HPV infection showed a generally upward trend over time. The year with the largest number of publications was 2020 with 43 articles published, followed by 2019 and 2014 with 36 publications, 2018 and 2013 with 29 publications.

The number of articles published each year ranged from 2(in year 1994) to 43(in year 2020). According to their abstracts, the first publication in our collection that explicitly addressed the co-infection phenomenon of different HPV genotypes of HPV was in 1994. The study showed the occurrence of HPV16 co-infection with HPV31/33/35 detected in 234 cervical specimens from 1126 Alaska Native women (30). In our collection, the first article that involved epidemiological analysis with HPV multiple infections in an epidemiological analysis was published in 1999 by Chan et al. (17) indicating that doubleual HPV infection was more frequently detected with a significantly higher proportion in patients with normal or inflamed cervices than those with cervical intraepithelial neoplasia(CIN) or carcinoma.

### Analysis of journal and impact factor

The 463 publications were from 200 different journals with article on HPV multiple genotype co-infection published. Overall, 33.5% (67/200) journals published more than two documents.

**Figure 2:**
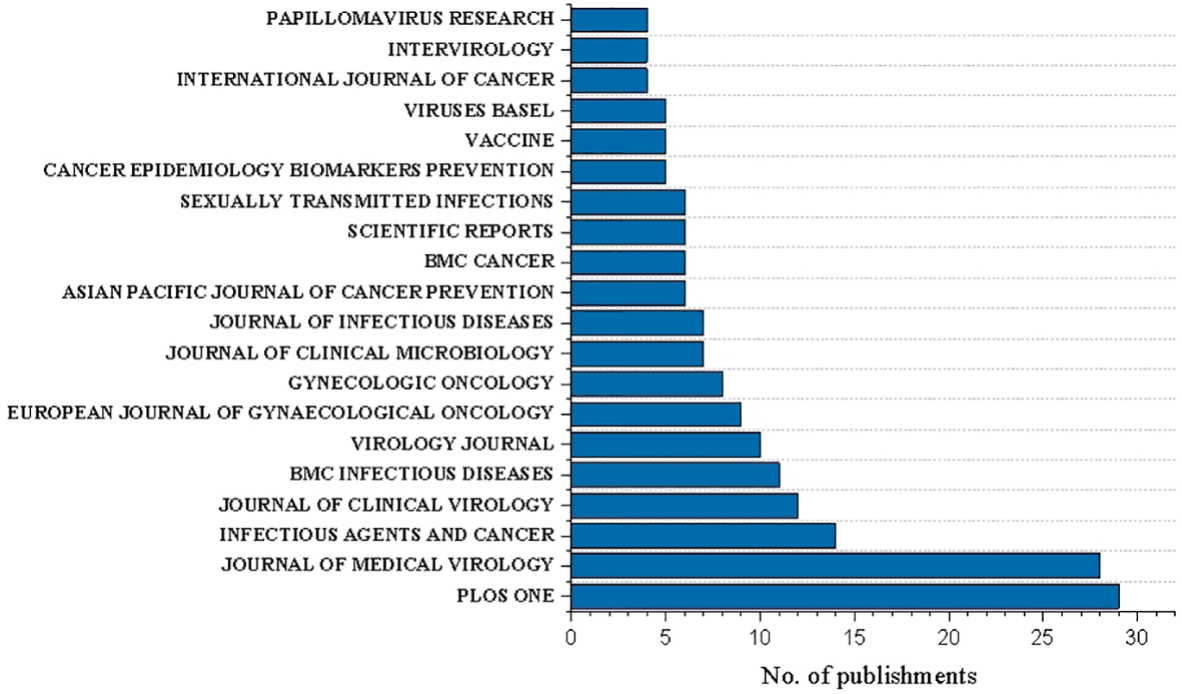
Number of publications on HPV co-infection in the top 20 journals.

The most represented journals were PLOS ONE (n=29, Five-year impact factor: 4.069), JOURNAL OF MEDICAL VIROLOGY (n=28, Five-year impact factor: 12.203), and INFECTIOUS AGENTS AND CANCER (n=14, Five-year impact factor: 3.572), JOURNAL OF CLINICAL VIROLOGY(n=12, Five-year impact factor: 7.85), BMC INFECTIOUS DISEASES(n=11, Five-year impact factor: 3.714) (**Figure 2**).

### Analysis of country, author and organization

**Figure 3:**
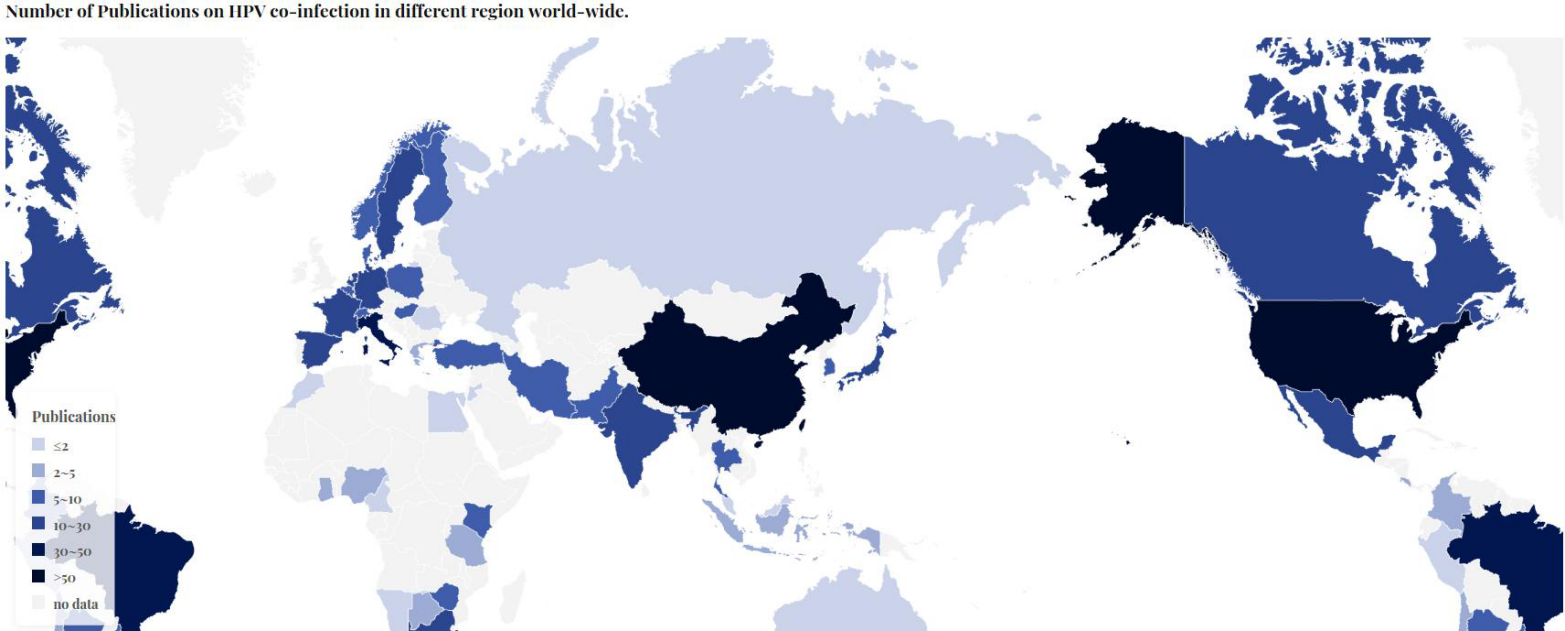
Number of publications on HPV co-infection in different countries and regions.

The literatures studied were from 78 countries and regions. The United States(n=73) led the way in this category, followed by China (n=65) and Italy (n=50). In Europe, France and Spain also made significant contributions to this research field, with 28 and 29 articles respectively (**Figure 3**).

In our literature collection, a total of 89 researchers contributed more tha 3 articles. Villa LL, the author with the most publications contributed, participated in 8 relevant literatures, among which the most cited publication focused on the persistent infection of HPV, believing that HPV co-infection was an important factor for the prolonged duration of infection (16). One author (Tommasino M) has contributed to seven articles, and another(Gheit T) has contributed to six articles. Chan PKS, Gardella B, Picconi MA, Schmitt M, Spinillo A and Williamson AL contributed to the publication of five papers.

In our collection, 46 institutions contributed more than 5 articles to the publication of literature, and the top 5 contributing institutions were UNIVERSIDADE DE SAO PAULO(n=14), WORLD HEALTH ORGANIZATION(n=13), INTERNATIONAL AGENCY FOR RESEARCH ON CANCER IARC(n=12), KAROLINSKA INSTITUTET(n=10), DDL DIAGNOSTIC LABORATORY(n=9), INSTITUTO MEXICANO DEL SEGURO SOCIAL(n=9), CHINESE ACADEMY OF MEDICAL SCIENCES PEKING UNION MEDICAL COLLEGE(n=8), UNIVERSITY OF CAPE TOWN(n=8), VRIJE UNIVERSITEIT AMSTERDAM(n=8), FUNDACAO OSWALDO CRUZ(n=7).

### Analysis of keyword co-occurrence cluster

**Figure 4:**
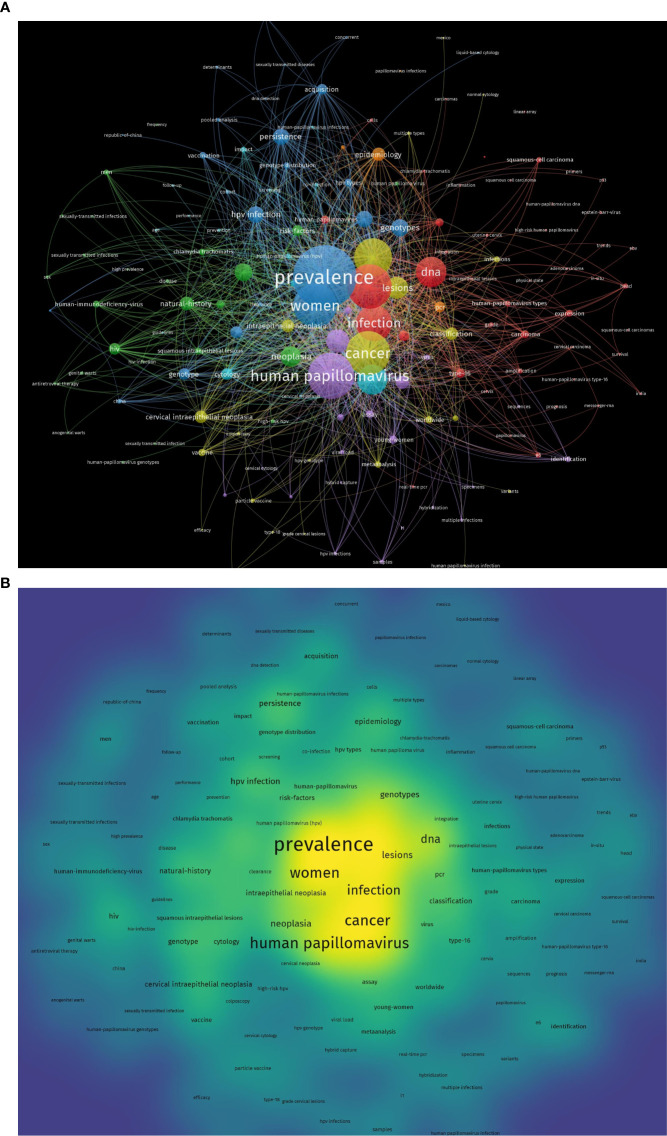
Network plot of keywords in the publications collection of HPV co-infection. **(A)** Network visualization. **(B)** Density visualization.

The co-occurrence keywords network visualization map with extraction frequency more than five times or more was shown in **Figure 4**. There were 158 high frequency keywords extracted from 463 articles, which can be classified into 7 clusters listed below. Cluster1: ‘human papilloma-virus’(red color) including included 45 items such as DNA, cells, cervix, carcinoma. Cluster2: ‘neoplasia’(green color) including included 27 items such as cervical lesions, frequency, HIV, hpv genotype, hpv prevalence. Cluster3(blue color): ‘prevalence’ including included 25 items such as co-infection, concurrent, genotype distribution, hpv infection, hpv types, vaccination. Cluter4:’cancer’(yellow color) including included 23 items such as risk, lesions, multiple infections, cervical intraepithelial neoplasia. Cluster5:’human papillomavirus’(purple color) including included 21 items such as diagnosis, cervical cytology, multiple infections, sequences, virus, samples. Cluster6: ‘cytology’ including included 13 items and cluster7 includes included 4 items.

## Discussion

*The high incidence of HPV co-infection is still being updated by epidemiologists around the world. In the latest epidemiological statistics published in 2023, high rates of HPV polygenotype co-infection have been reported in Asia, Africa, and South America (* 31–33)*. Although HPV polygenotypes infecting the same individuals have long been speculated to be a possible symbol of cervical cancer progression and a less optimistic prognosis, there has been no conclusive evidence to confirm this until now. In the study conducted by Maria Teresa Bruno et al on the correlation between HPV single infection and multiple infection and clinical pathogenesis (2020), the probability of CIN1 in a single infected person is 39.5%, which is significantly lower than the probability of CIN1 in multiple infected persons (60.4%). However, the opposite result is shown in patients with cervical cancer (* 34)*. This may indicate that multiple infections are more likely to stimulate the occurrence of early cervical lesions, but lack high carcinogenic effect. Zeni Wu et al. also pointed out the similarities and differences in expression of oncoprotein in single and multiple genotypes pattern through detection of E6 oncoprotein in single and multiple genotypes pattern (2019)* (35)*. All these studies have opened a new idea for clinical research on HPV multiple infection. Through in-depth learning of HPV multiple infection, it may have crucial clinical practical significance for the treatment of cervical diseases, the development of HPV genotype-specific vaccines, and the improvement of cervical cancer prognosis.*

According to our investigation on highly cited article collections, studies on infection mechanism, clinical practice and Epidemiological characteristics have became the top three research direction in the number of citations. In the top 20 cited articles, a study published in 2011 (11) which analyzed the mechanism of co-infection on the basis of epidemiological data, suggested that the infection between different HPV genotypes might happened randomly, denying interaction between thoes HPV genotypes. However, another research published in 2010 (19) indicating that the tendency for specific HPV genotype to cluster increased with the genetic similarity of the L1 region. About disease progression and clinical practice, a study published in 2004 (2) with the most citation number(n=225) pointed that younger women were significantly more likely to harbour multiple high risk HPV infections, but multiple HR-HPV infections were not more frequent in high grade than in low grade cervical neoplasia. The most cited study on HPV testing technique is a 2008 paper published on J CLIN MICROBIOL with a conclusion that BSGP5+/6+-MPG is suitable for epidemiologic and also diagnostic applications (13). The analysis of research themes on the highly cited literatures can help us understand the main focus of researchers on HPV co-infection at this stage, indicating that the in-depth exploration of previous work in these directions is more informative for future researchers.

Changes of the numbers of publications and citations over the years can also give us some insight and conjectures. The year in which the highest amount and quality studies of HPV co-infection were published can be observed in the number of citations over time. In this article, we found that there was a peak in the total number of citations around 2006, which we suspect might be related to the world’s first FDA approval of the HPV vaccine in 2006. However, with the HPV vaccine being put into clinical practice in most countries around the world, the number of publications related to HPV infection has decreased significantly in the past five years until 2010, indicated that since human beings have developed better coping strategies in face of HPV infection and are closer to the goal of eliminating HPV infection, the research motivation seems to be reduced. When it comes to 2009, German scientist Harald Hausen was awarded the Nobel Prize in Physiology and Medicine for his discovery of a causal relationship between HPV and cervical cancer, which was seen as the basis for further vaccine research. We suspect that the steady increase in the number of publications on HPV co-infection which began after 2009 possess certain relevance to this issue, as this Nobel Prize gave significant recognition to the clinical value of all HPV-related research. Significant scientific accomplishments and major events in the field have never failed to stimulate research enthusiasm, and HPV research has evolved to the point where the scope of research should be narrowed down and discover more unknown phenomena rather than known.

Statistically, JOURNAL OF MEDICAL VIROLOGY ranked among the top five publications with the highest impact factor, which reached 12.203 in the past five years. In this journal, a total of 28 literatures related to HPV co-infection were published, most of which focused on the geographical distribution of HPV infection. Studies related to the epidemiology of HPV co-infection have been in-depth and detailed to a certain extent, but the number of higher quality literature published in mainstream journals is far from enough. Higher impact factors and more authoritative publications mostly equate to more informative and influential accomplishments and discoveries. At this stage, although HPV co-infection research has gained some results in simple analysis of large sample data collection, there are still few people who can explore the essence through the phenomenon and lack higher-level interpretation of the mechanism. As expected, HPV genotypes co-infection remains a niche topic that has received less attention and investment in this area.

The analysis of the number of studies by country and region also allows us to draw some reliable conclusions. Among the data we obtained, the United States and China contributed the highest number of publications, and in addition to their scientific strength, the significant population base of these two countries is an important factor that cannot be ignored. Among the European countries which has relatively small populations, Italy, France, and Spain also ranked among the top, indicating that there is also a great interest and concern in the topic of HPV co-infection in western developed countries. The number of publications was lowest in less developed regions such as Central Asia, South America and Africa. Based on some epidemiological statistics, there is also a correlation between HPV infection rates and ethnicity, so it is crucial that countries around the world work together and share findings to further our understanding of this virus comprehensively.

The results of the keyword co-occurrence analysis of our collected articles suggest some of the most frequently occurring keywords in the existing studies. In **Figure 4**, it is easy to recognize that ‘Prevalence’ is the most frequent keyword, and the keywords ‘Population’, ‘genotype’, ‘Pcr’, and ‘Sequences’ also appear frequently. This suggests that epidemiological studies are still the main research direction for HPV co-infection, and this overlaps with other evidence-oriented findings in this paper.

The concept of HPV co-infection was first mentioned in the 1990s, and at that time it only remains a simple description of this phenomenon without any statistical description. With the improvement of sophisticated HPV genetic testing technique, more HPV genotypes can be detected and the major genotype combinations of HPV infection are gradually recognized. When the time came to around 2010, the literature on HPV epidemiology studies no longer ignored co-infection and the exploration of co-infection pattern even infection mechanism went much further. *Mathematical models have recently been studied to understand the transmission dynamics of HPV infection (* 36)*, the construction of the co-infection mathematical model of HPV and other virus benefits for the control and management of the disease (* 37, 38)*. In the future, the establishment of co-infection models exclusively involving multiple HPV genotypes may yield valuable insights.* Although the amount of existing article is still insufficient, the current article can be used as the first step into this field, following the top journals, authors and publications, setting the stage for in-depth discussion.

This study also has its limitations. First of all, in terms of citations statistics, some articles published in recent years need more time to accumulate citations, so there may be some inevitable bias in the analysis of citations. The keyword search strategy of Web of Science is difficult to filter out publications containing “HPV” and “coinfectoin” but focused on HPV coinfecton with other viruses, resulting in the total number of calculated articles exceeding the actual number. Also, due to the search strategy limitations, HPV co-infection mentioned in this article has not distinguished between high-risk HPV, low-risk HPV and potentially high-risk HPV. Restrictions on the language of inclusion may result in some articles written in other languages not being included. *Last but not least, since this bibliometrics research is conducted by using the web of science Core collection as the archive, relevant articles that are not included in the core collection or belong to other repositories may inevitably be omitted. Despite the fact, this article might still be the largest bibliometric study on the topic of HPV polygenotypic co-infection.*

## Data availability statement

The original contributions presented in the study are included in the article/supplementary material. Further inquiries can be directed to the corresponding authors.

## Author contributions

Conception and design: YL. Collection and assembly of publications: TB. Manuscript writing: TB/YG. Final approval of manuscript: All authors. All authors contributed to the article and approved the submitted version.
